# No evidence of inbreeding depression in a Tasmanian devil insurance population despite significant variation in inbreeding

**DOI:** 10.1038/s41598-017-02000-y

**Published:** 2017-05-12

**Authors:** Rebecca Gooley, Carolyn J. Hogg, Katherine Belov, Catherine E. Grueber

**Affiliations:** 1 School of Life and Environmental Sciences, University of Sydney, Sydney, NSW 2006 Australia; 2 Zoo and Aquarium Association Australasia, Mosman, NSW 2088 Australia; 3 San Diego Zoo Global, PO Box 120551, San Diego, CA 92112 USA

## Abstract

Inbreeding depression occurs when inbred individuals experience reduced fitness as a result of reduced genome-wide heterozygosity. The Tasmanian devil faces extinction due to a contagious cancer, devil facial tumour disease (DFTD). An insurance metapopulation was established in 2006 to ensure the survival of the species and to be used as a source population for re-wilding and genetic rescue. The emergence of DFTD and the rapid decline of wild devil populations have rendered the species at risk of inbreeding depression. We used 33 microsatellite loci to (1) reconstruct a pedigree for the insurance population and (2) estimate genome-wide heterozygosity for 200 individuals. Using heterozygosity-fitness correlations, we investigated the effect of heterozygosity on six diverse fitness measures (ulna length, asymmetry, weight-at-weaning, testes volume, reproductive success and survival). Despite statistically significant evidence of variation in individual inbreeding in this population, we found no associations between inbreeding and any of our six fitness measurements. We propose that the benign environment in captivity may decrease the intensity of inbreeding depression, relative to the stressful conditions in the wild. Future work will need to measure fitness of released animals to facilitate translation of this data to the broader conservation management of the species in its native range.

## Introduction

The Tasmanian devil (*Sarcophilus harrisii*), the largest extant carnivorous marsupial, faces extinction due to the emergence of a contagious cancer called Devil Facial Tumour Disease (DFTD)^1^. In 2006, Australia’s largest insurance metapopulation was established under management of the Save the Tasmanian Devil Program (STDP), in collaboration with the Zoo and Aquarium Association (ZAA), to breed Tasmanian devils away from the disease for ultimate release back into the wild^2^. Preservation of the genetic diversity of the species is critical and, at its establishment, the insurance population aimed to maintain 95% of genetic diversity over 50 years. Within the Tasmanian devil insurance population (and also in many other insurance populations for other species) the relationship of the founding individuals was unknown.

The insurance metapopulation was seeded by a total of 122 founders collected over four intakes from 2005 to 2008^3^. Young, dispersing juveniles were sourced and combined with the existing captive population of 112 animals, originating from 25 genetic founders^4^ (for a review and map of recent and pre-existing founder provenance see ref. 2). At the time of collection, the rapid spread of DFTD across the island of Tasmania resulted in devils being preferentially sourced from the north-west of Tasmania, as populations there were disease-free (see ref. 3). Despite best efforts, it is possible that some of the initial founding animals were closely related (accounting for dispersal patterns of Tasmanian devils and founder collection locations^2, 3^). As founder intakes were weighted to two specific regions (see map^2^), and Tasmanian devils show minimal genetic differentiation even within regions^2, 5^, we believe the pedigree of captive Tasmanian devils may not reflect all true relationships. Molecular data is consistent with trapping data of individuals in close proximity (this study).

The Tasmanian devil insurance population is managed using a traditional mean kinship approach^6^ based on pedigree analysis. This approach is successful in minimizing the overall kinship of a population, preventing or limiting inbreeding and retaining genetic diversity^7^. Founding individuals, by necessity, are assumed unrelated and not inbred, as information from the wild is generally unavailable. Rudnick & Lacy (2008) explored the impact of founder relationship assumptions on the success of mean kinship management using simulations. The long-term consequences (over 5 generations) of assuming founders to be unrelated were small when compared to a model of known founder relationships and known inbreeding values. However, short-term consequences (3 to 5 generations) of an initial increase in inbreeding values were exposed when founders were assumed to be unrelated compared to a model of known founder relationships and inbreeding^7^. This finding highlights the importance of understanding the recent ancestry of founders when commencing an insurance population, particularly those which will be used for rewilding and genetic rescues soon after instigation, such as in the case of the Tasmanian devil insurance population.

Genetic diversity of the Tasmanian devil is low^8, 9^. Tasmanian devils were previously found on mainland Australia approximately 5,000 years ago, but are now restricted to the island state of Tasmania^10^. At least three population crashes, resulting in genetic bottlenecks, have been documented in Tasmania since devils were isolated on the island approximately 10,000 years ago^11^. Most recently, this species has suffered an extreme population decline (~85%)^12^ due to the emergence of two transmissible cancer lines, Devil Facial Tumour 1 (DFT1) and Devil Facial Tumour 2 (DFT2), first documented in 1996 and 2015 respectively^13, 14^. These severe population declines have rendered the Tasmanian devil at risk of further genetic diversity loss and inbreeding depression.

Inbreeding depression is the decrease in individual fitness that occurs as a result of increased homozygosity via inbreeding. The scale of the Tasmanian devil insurance population, and access to genetic samples from the entire population, provides a unique opportunity to explore the effects of inbreeding on fitness in an intensively managed, newly established population. Inbreeding depression is well documented in both captive and wild populations across numerous taxa (e.g. refs 15 and 16) and can vary widely both among and within species, for multiple reasons (e.g. founding population size and diversity^17, 18^ or available resources^16^). This can create a dilemma for captive breeding programs, as the general strategy for management is to minimise mean kinship. However as each species program and life-history varies an understanding of the relationship between inbreeding, life-history and management practice may be needed to minimise the negative effects of inbreeding.

The detection and evaluation of inbreeding, and inbreeding depression, can be achieved through two different approaches: pedigree-derived inbreeding coefficients, or molecular-based inbreeding estimates. The relationship between these two metrics and the relationship they independently have with individual-fitness measures have differing strengths and weakenesses^19–22^. Wright (1992) provided a method to calculate a coefficient of inbreeding, F, using pedigree information. This approach relies on two assumptions; (1) founding individuals of the pedigree are unrelated, and (2) the pedigree information is an accurate representation of genetic relationships and degree of shared ancestry. If portions of the pedigree are unknown, or the relationship between recent founders is unknown, pedigree-derived inbreeding values inaccurately represent true variation in genetic relationships. Molecular-based inbreeding values offer an alternative to pedigree-based values, by providing a multi-locus heterozygosity score intended to be representative of genome-wide inbreeding levels^22^. This technique operates predominately under the expectation that increased inbreeding will increase the probability that an individual is homozygous for deleterious recessive alleles, leading to inbreeding depression^20, 21^. This approach however can be limited by sampling variance, such that multi-locus heterozygosity at the markers used may not be representative of genome-wide heterozygosity. Nevertheless, molecular measures of inbreeding enable heterozygosity-fitness correlations (HFCs) to be used as a tool for detecting inbreeding depression in individuals with limited or unknown pedigree information.

HFCs may be based on a wide range of direct and indirect measures of fitness, generally categorised as direct measures of fitness, including life-history traits (survival and reproductive success), versus indirect measures of fitness, including physiological (e.g. parasitic load) and morphological (e.g. body weight) traits^19^. On average, slightly stronger HFCs are detected when using direct fitness measures^19^. Significant HFCs are still detected using indirect fitness correlates^23–25^, and these measures are often more easily attainable than direct measures, such as survival of long-lived species, or reproductive success in wild populations. In the presence of inbreeding depression, HFCs may not be detected unanimously across all measures of fitness (e.g. refs 26 and 27). Thus, studying both direct and indirect mechanisms by which inbreeding depression affects various traits in a single species is necessary. Identifying these traits can help conservation research more broadly.

Ultimately, the detection of HFCs can only take place when there is variance in inbreeding among the study population^20, 28^. Variance in inbreeding within a population can be detected as identity disequilibrium. Using a multi-locus approach, covariance in heterozygosity across markers can be used to measure identity disequilibrium and quantify the variance in inbreeding^21, 29^. In endangered species, particularly those in captive breeding programs derived from restricted founding populations, the likelihood of identity disequilibrium is high.

In this study, we investigated whether a captive population (part of the Tasmanian devil insurance metapopulation) showed signs of inbreeding depression across six measures of fitness using both pedigree-based and molecular-based approaches. We developed microsatellite markers to enable pedigree reconstruction of devil juveniles produced in group-breeding enclosures (multiple males and females housed together), and examined whether the pedigree or molecular data are informative of inbreeding depression at six fitness traits, including direct and indirect measures of fitness, in captivity.

## Methods

### Study Species and Data Collection

This study was conducted at a Tasmanian devil breeding facility located in NSW, Australia. The facility consists of 12 managed environmental enclosures (MEEs) and eight intensive enclosures built over 25 hectares. In MEEs, Tasmanian devils are housed at a density of approximately 2.67 devils/hectare, consisting of eight 3-hectare enclosures holding 4:4 (male:female) devils and four 2.5-hectare enclosures holding 3:4 devils^30^. Intensive enclosures generally house 1–2 individuals, either for welfare reasons or breeding purposes. Breeding recommendations are issued annually and not every devil is provided with a breeding opportunity every year. The breeding facility was established in January 2011 with 30 devils (11 wild founders^3^, 2 hand-raised orphans, and 17 captive born devils from the existing zoo insurance population^2^). A limited number of transfers into the facility occurred in subsequent breeding seasons (fewer than 10 devils per year). However, 26 of the transferred individuals were related (litter mates), comprising 10 litters.

Tasmanian devil females give birth to multiple offspring (20–30), but have only four teats in their pouch and as a result a maximum of four offspring can survive per female per breeding season^31^. Mixed paternity litters are possible. The breeding season occurs between March to June^32^. Sampling for this study occurred during the annual catch up event over a three-week period during November and December in 2014 (under approval from University of Sydney Ethics Committee #2014/550). Following approved ethical standards, Tasmanian devils were restrained in a hessian bag and ulna length, asymmetry and testes volume were recorded (full measurement methods are below). As part of regular husbandry practices (following approved ethical standards), reproductive outputs and weight at weaning were recorded for all Tasmanian devils from 2011 to 2014.

Due to the nature of fitness measurements collected, six datasets were identified for statistical analysis. Table 1 describes the fitness measurement collected for each dataset and the corresponding sample size. In total, 200 Tasmanian devils were included in this study.

**Table 1:** Summary of Tasmanian devil fitness measurements collected for the six datasets used in this analysis.

Dataset	Measurement^†^	n (m:f)	Mean (±SD)
1	Ulna length^1^	91 (43:48)	12.45 cm (0.73)
	Asymmetry^2^		0.11 cm (0.14)
2*	Testes volume^3^	43	10.09 cm^3^ (2.81)
3	Weight at weaning^4^	159 (64:95)	4.66 kg (1.20)
4	Female reproductive success^5^	88	1.20 offspring (1.43)^‡^
5	Male reproductive success^5^	87	1.27 offspring (1.61)^‡^
6	Survival^6^	197 (90:107)	5.1 years (1.66)^§^

*Male subset of dataset^1^.

^†^Superscripts ^1–6^correspond to phenotypic traits described in Supplementary Methods.

^‡^Reproductive mean includes non-breeders.

^§^Survival mean calculated only from deceased Tasmanian devils during this study period (although all devils were included in the analysis, as reflected by n).

### Phenotypic Traits

Six phenotypic traits were measured (Table 1), four indirect fitness measures and two direct fitness measures. All indirect measures were morphometric: (1) ulna length, a measure of individual size, can affect both competition and (indirectly) reproductive success (e.g. ref. 33), (2) asymmetry, a measure of development stability, can affect individual welfare and performance across a range of species (for a review see ref. 34), (3) testes volume, which can correlate with individual reproductive output in mammals (e.g. ref. 35) and (4) weight at weaning, which can influence adult body weight in marsupials and may influence survival (e.g. refs 36 and 37). Direct measures of fitness were life-history traits (5) reproductive success and (6) survival. For a full description of the specific measurements, see Supplementary Methods.

Each fitness trait was analysed independently for inbreeding depression in this study. Along with the above-mentioned fitness measurements, each individual had the following data recorded: age, sex, year, pen total (the number of individuals housed within the same enclosure) and pen ID. This data was obtained from studbook information^38^ and annual housing reports from the breeding institution.

### Marker Development, DNA Extractions and Molecular Measurements

DNA samples had been previously extracted from ear biopsies and blood samples collected as part of the management of the Tasmanian devil insurance population^39^ with further samples collected in 2014 under the direction of the ZAA, on behalf of the STDP, for management purposes. Samples were extracted using a modified phenol-chloroform extraction technique^40^ and stored at −20 °C. All individuals were genotyped for 33 microsatellite markers (Table 2).

**Table 2:** Characteristics of the 33 microsatellites used to genotype Tasmanian devils.

Locus (Chromosome)	Multiplex	Fluorescent label	Primer sequences (5′-3′)	Repeat motif	Na	Repeat Length	*n*	Ho	He
Sha034*^(Chr 6)^	M1	6-FAM	F-TGGAAAGAACTGATGAACAG	(AT)_10_AC(AT)_6_	3	221–225	199	0.250	0.253
			R-TGTACATGAAATTCCAAGTC						
Sha011*^(Chr 1)^	M1	NED	F-CATTCTTGCACATACATGTC	(CA)_16_	2	242–244	200	0.139	0.154
			R-GATTCAACTATTCTGGAGAG						
Sha036*^(Chr 4)^	M1	VIC	F-TTGTAGAATGAAGTGGAGTG	(AC)_21_	3	314–318	198	0.441	0.412
			R-CTGATTGTTCTCACATTGTG						
Sha042*^(Chr 4)^	M2	6-FAM	F-TTCCCATTACAGTCCAGGTG	(AC)_15_	2	211–215	200	0.525	0.500
			R-GGCAGACAGGGTTAAGTGAC						
Sha032*^(Chr 6)^	M2	PET	F-TAGTTGTATGGTTACCTGAG	(TA)_2_CATACA(TA)_14_	4	310–316	151	0.159	0.149
			R-CGTGATAGTTATTATATGAG						
Sha014*^(Chr 1)^	M2	NED	F-AGGTATGAAGGTAGGAAGAG	(CA)_9_TA(CA)_16_	5	344–354	199	0.520	0.584
			R-TAATCTGGGCTGGTAGTAGG						
Sha023*^(Chr 2)^	M3	PET	F-CACTTCTGGCATATTATCTG	(TA)_5_CA(TA)_14_	7	268–280	178	0.554	0.663
			R-TGGTTATTACATTCATTGAG						
Sha001*^(Chr 2)^	M4	6-FAM	F-GCAGCTATGTACACAAACTC	(TC)_22_	3	226–230	200	0.441	0.466
			R-GGGCTCATCTAGAGGTC						
Sha009*^(Chr 4)^	M4	PET	F-TTTCACTACCTATGTCACTG	(CA)_21_	2	244–246	200	0.267	0.246
			R-ATTTATCAGCATCAGGAGAC						
Sha013*^(Chr 1)^	M4	6-FAM	F-AGGGAGACTGCCATCTAGTG	(TC)_32_	7	292–308	199	0.731	0.737
			R-CATCTTACAACTTGTGTGAC						
Sha025*^(Chr 2)^	M5	VIC	F-GAATAGACAACTCTTTACTC	(TG)_22_	2	236–238	191	0.449	0.484
			R-GTACAGCTAGGATTGTGGAC						
Sha010*^(Chr 4)^	M5	PET	F-TCTATCATTGATTGGGTCCT	(AT)_19_	9	324–358	198	0.750	0.739
			R-ACGATGACTGAAGCTGACTC						
Sha028*^(Chr 5)^	M6	NED	F-TTCATTACAATATTCAAGAG	(AT)_14_AATATGC(AT)_3_	5	171–179	168	0.610	0.632
			R-CAAACATAAACAAGTGAGAC						
Sha039*^(Chr 4)^	M6	PET	F-CAGAGTTACACAAATGTCAC	(TA)_12_	4	169–177	198	0.333	0.380
			R-AGCATGAGAATTTGGAAGTC						
Sha040*^(Chr 4)^	M6	6-FAM	F-TGACTGACTGCCAAGTGGAC	(AC)_2_A(AC)_17_	4	177–193	199	0.640	0.665
			R-TGCCAGCAAATCATCTAACC						
Sha012*^(Chr 1)^	M6	VIC	F-TCCAATTCAGTACTCTATCC	(TG)_25_	5	183–197	184	0.383	0.368
			R-TGGCATTTAATGATCTCTAC						
Sha037*^(Chr 4)^	M6	NED	F-ATGAATCCAGGGTTCTACTC	(TA)_22_	7	200–214	198	0.653	0.653
			R-GGTATCTGTCCTCAAGAAGC						
Sha008*^(Chr 2)^	M6	VIC	F-AGTGTGGGAAAGCTATAGAG	(AC)_22_	5	241–251	199	0.540	0.510
			R-AATAATTGGGTGATGAGTCC						
Sha026*^(Chr 2)^	M7	VIC	F-CCAGGGCTCTATTCACTGAG	(GT)_3_AT(GT)_21_	4	269–277	199	0.487	0.541
			R-CTTCATATTTGCCATTTCTG						
Sha024*^(Chr 2)^	M8	VIC	F-TTCTAAGAGATGATACTACC	(AC)_18_	3	187–191	184	0.268	0.291
			R-CTTCAGGAGATTATTATGAG						
Sha015*^(Chr 1)^	M9	6-FAM	F-AATATTTGACTGCTATACTG	(TGAA)_6_	2	306–310	198	0.480	0.499
			R-ATCCACTTTGCCACTGTACC						
Sha033*^(Chr 6)^	M9	NED	F-TCTCACATGTACCCTCACAG	(AC)_21_ATAT(AC)_4_	4	323–329	199	0.295	0.313
			R-TGTTTCACTCTTGCCATCTG						
Sh2v^†^	M1	PET	F-TTGGAGAAAATGGAAGCAG	(AC)_23_	8	187–205	198	0.615	0.665
			R-CAGGATCTATTTTCTGAGTTAAGG						
Sh2b^†^	M3	NED	F-GCTCAGCACTTCCAGCCTTG	(CA)_20_	2	112–116	199	0.253	0.259
			R-GAAGCAAGTTTCCCAAGAGGTG						
Sh2i^†^	M3	6-FAM	F-GCTACTGCGGAGTCAGATTGC	(CA)_20_	3	227–231	199	0.567	0.517
			R-GAAGTATACGTCTGCTATGTCCC						
Sh2L^†^	M4	VIC	F-ACACTCCATGTTTTAGTTTG	(CA)_11_T(AC)_17_	3	173–177	200	0.168	0.228
			R-TCGGTATGTGTGTCTCTCAG						
Sh2g^†^	M5	6-FAM	F-CCTTTCAAAGCCACATCCTAAG	(AC)_21_	4	116–122	200	0.554	0.616
			R-TTGGTTTGATACTGGAGGACAG						
Sh6L^†^	M7	PET	F-AGATGGTCTGAGCATGTATCC	(CA)_4_(CT)_2_CCCTA(AC)_20_	3	145–149	196	0.415	0.448
			R-TAGTCCAGGTGTGAGGTGATG						
Sh3a^†^	M8	6-FAM	F-TGAACCCCAAGCTCTATCAG	(CA)_18_	2	186–188	200	0.530	0.494
			R-CTTCCCCTGTAAGTGTATTTG						
Sh6e^†^	M8	NED	F-GATTCTAGAAGGGATAGCAAGC	(CA)_6_(A)_2_(CA)_18_	2	202–204	197	0.437	0.384
			R-GACACTCCATAGAAATGCACTG						
Sh5c^†^	M9	VIC	F-CCCCATCTTATAATGAAAGTC	(CA)_16_CGCTCG(CA)_2_CG(CA)_4_	3	113–121	200	0.201	0.226
			R-ATCAGAAGCAACAAAACCAG						
Sh2p^†^	M9	6-FAM	F-TGCCCCATCACACTTTCCTTG	(CA)_18_	5	143–153	199	0.495	0.560
			R-GCAATCCTGGTCATGATGTAGTC						
Sh3o^†^	M9	6-FAM	F-CTCAATGCCAAAGGTATCTTC	(CA)_22_	4	224–230	200	0.589	0.527
			R-CATAGTTCCAAATCACTCTCCAG						

*Current study.

^†^Jones *et al*.^41^.

Na = number of alleles observed for microsatellites, H_o_ = observed heterozygosity and H_e_ = expected heterozygosity.

At the commencement of this study there were only 11 putatively-neutral microsatellite markers characterised in devil^41^ (Table 2). In order to assess HFCs, additional microsatellite marker development was required. Devil-specific microsatellite makers were developed using the Tasmanian devil genome accessible on Ensembl^42^. Microsatellite repeats were identified throughout the genome using RepeatMasker^43^. Genomic data processed in RepeatMasker was a minimum of 10 kb away from coding DNA and sourced evenly across all six Tasmanian devil autosomes. All candidate microsatellites were a minimum of 10 kb apart, to minimise the possibility of linkage disequilibrium. In total, 44 candidate microsatellites were chosen for polymorphism screening. Primer pairs were designed and optimised using Oligo 7 (Molecular Biology Insights). Loci were initially screened for variation using 12 Tasmanian devils, randomly selected from the insurance population. For the initial screening process, forward primers were labelled with a universal 6-FAM CAG Tag (CAGTCGGGCGTCATCA) (following^44^). PCRs were performed following the standard protocol for the Qiagen Type-it Microsatellite kit, with modified primer concentrations of 0.06 µM for tagged primers and 0.6 µM for untagged primers and the CAG tag. Thermocycling conditions followed a protocol of 95 °C for 5 min, followed by 30 cycles of 95 °C for 30 s, 60 °C for 90 s and 72 °C for 30 s with a final extension of 60 °C for 30 min. Twenty-two polymorphic microsatellites were identified (Table 2): five on autosome 1, six on autosome 2, seven on autosome 4, one on autosome 5 and three on autosome 6 (5 and 6 had fewer markers due to the relatively smaller size of these autosomes compared to autosome 1–4; all candidate microsatellites on chromosome 3 were found to be monomorphic after screening across 12 Tasmanian devils). See Supplementary Table S1 for monomorphic microsatellites.

For genotyping all animals included in this study, the forward primer of each marker was fluorescently labelled (Table 2). PCRs were performed following the standard protocol for the Qiagen Type-it Microsatellite kit, with the modification that the reaction size totalled 10 µL. Fragments were separated on an ABi3300 using GeneScan™ 500 LIZ™ as size standard. Nine multiplexes were developed using Multiplex Manager^45^ (Table 2). Genotypes were scored using GENEMARKER (SoftGenetics, State College, PA). A randomly chosen group, comprising 16.5% of all individuals, were re-genotyped to estimate genotyping error rate. Results were screened for null alleles using MICROCHECKER^46^. GenAlEx^47, 48^ was used to calculate observed (H_o_) and expected (H_e_) heterozygosity, and number of alleles, for each locus (Table 2). Hardy Weinberg Exact tests were conducted with Genepop^49, 50^.

Internal relatedness (IR) was calculated as a measure of individual multilocus heterozygosity, using the *Rhh* ^51^ package in R^52^. The IR metric gives higher weighting to rare alleles in the population, making this method ideal for studying inbreeding^53^. Homozygous individuals for a rare allele will have a higher IR value than homozygous individuals for common alleles, due to the increased chance that rare allele homozygosity is the product of an inbred mating^53^. There are numerous methods to calculate multilocus heterozygosity, a commonly used alternative being standardised multilocus heterozygosity. In our dataset, the correlation between IR and standardised heterozygosity was 0.908. As the correlation was high and IR is most suitable for studying inbreeding^19^, we selected IR for subsequent analyses.

### Pedigree Construction

Parentage (paternity and maternity) of offspring produced in group pens was assigned using CERVUS^54^, using a combination of molecular data and annual enclosure records of the location of each Tasmanian devil. Paternal and maternal candidates included all sexually mature Tasmanian devils present at the institution during the breeding season for each offspring cohort. A total of 118 joeys were born during the years 2011–2014 (2011 n = 24, 2012 n = 38, 2013 n = 29 and 2014 n = 27); all of which were genotyped in this study. All inferred parent-pair combinations were checked against annual reports and only accepted if individuals had been present in the same enclosure during the reproductive season. Critical LOD scores were calculated for each cohort by simulation of 10,000 offspring at an error rate of 1% (proportion of loci mistyped = 0.01). LOD confidence calculations were set at strict confidence = 95% and relaxed confidence = 80%. All candidate parents were analysed using joint LOD scores to assign parentage. All genetically confirmed parents (i.e. known parents) had 0 mismatches with their offspring. All remaining potential parent pairs had >3 mismatches, and were therefore confidently excluded as potential parents for a given offspring. Known parentage data (genetically assigned parents) was entered into the studbook and pedigree analysis undertaken using the PMx software^55^.

### Inbreeding Analysis

Identity disequilibrium can arise after, among other reasons, inbred matings, and result in heterozygosity and homozygosity correlations throughout the genome, within a population (reviewed in ref. 21). To assess whether a chosen panel of markers (number and type) inform individual inbreeding status the markers should be representative of variation in identity-by-descent (IBD)^29, 21^. Pedigrees may fail to detect variance in inbreeding due to unknown founder relationships, while molecular measures of IBD can expose inbreeding where it previously would not have been detected. Statistical evidence of identity disequilibrium provides support that a chosen marker panel measures variation in identity-by-descent and therefore may be useful for interpreting heterozygosity-fitness correlations under inbreeding^21^. Two analyses are commonly used to quantify the degree of identity disequilibrium, heterozygosity-heterozygosity correlations (HHCs)^56^ and the *g*_*2*_ statistic^57^. Both methods involve quantifying the degree of covariance in heterozygosity among markers to test for identity disequilibrium; we used both approaches here. We calculated *g*_2_ with the RMES software^57^, using 1000 permutations. The R package *Rhh* was used to calculate HHC using 1000 Monte Carlo simulations.

### Statistical Analysis

Our general approach used generalised linear mixed models (GLMMs) to assess the impact of internal relatedness (our predictor of interest) on five of our measures of fitness: ulna length, asymmetry, reproductive success, weight at weaning and testes volume (survival was modelled differently, see below). Mixed models were fitted using the R package *lme4* ^58^, where our indirect fitness traits of ulna length, asymmetry, weight at weaning and testes volume were fitted with *lmer* (Gaussian response variables) and our direct fitness traits (reproductive success) were fitted with *glmer* (binomial response variables). Fixed predictors in each global model included IR, as well as a unique set of fixed and random factors as relevant to the biology of each trait and each dataset (see below; full model specifications provided at Supplementary Table S2). Each global model was standardised to facilitate comparison of parameter estimates across models using the *arm* package^59^ in R. Model selection proceeded under information theory (following^60^). Briefly, we created a complete subset of models using the *MuMIn* package^61^ in R, submodels were ranked using the Akaike’s information criterion (corrected for sample size; AIC_C_), and model averaging was used to take all models that fell within 2 AIC_C_ of the highest ranked model. Inference was based on standardised effect sizes, their standard errors, and the relative importance scores for each included parameter^60^. This method can result in the exclusion of IR as a predictor from the final model, if its explanatory power is low. Under inbreeding depression, we predicted IR to be negatively correlated with testes volume, asymmetry, ulna length, weight at weaning, reproductive success and survival.

### Indirect Fitness Traits

In addition to the above general specifications, our indirect fitness trait models also included pen ID as a categorical random factor in each model (to account for between-pen variation). Our weight at weaning model also included “maternal IR” and “paternal IR” (in addition to the individual offspring IR) as continuous fixed predictors in the global model, due to the strong potential influence of parental effects on early juvenile traits. Specifically, the maternal investment provided to pouch young, and the molecular effects of both maternal and paternal internal relatedness on joey development. In the latter model, individuals were only included if all three IR values were available (joey IR, maternal IR and paternal IR). Global models for morphological traits also included sex (43:48 M:F), age and body weight (mean = 7.31 kg, SD = 1.62, n = 91) where appropriate (Supplementary Table S2). The age range of both male and females in this dataset spanned juveniles (1 year of age) to 5-year-old individuals. Tasmanian devils do not breed past the age of 5^32^ and pre-DFTD in the wild survived to 5 to 6 years of age^62^. They are known to live up to 9 years of age in captivity^63^.

### Direct Fitness Traits

Reproductive success for both females and males was modelled as a binomial process. For females, the number of events (successes) was the number of offspring produced by a female, and the number of trials was the total number of offspring biologically possible per breeding attempt (four potential joeys). For males, the number of events (successes) was the number of offspring sired by a male, and the number of trials was the total number of offspring sired by all individuals in the pen. Representing reproductive success as a proportional value in this way standardises across enclosures of differing population densities, sex ratios, and overall productivity (especially for males). Enclosure reproductive success was overdispersed for both sexes, whereby typically one male sired the majority of offspring in an enclosure, and approximately ~50% of females successfully reproduced in an enclosure. To account for overdispersion in our models we therefore added a residual parameter for each individual. In addition to IR, the male and female reproductive success models also included age as a continuous variable and pen ID and year as categorical random factors.

For both male and female devils the captive environment does not truly offer an opportunity for free mate choice, because males have limited opportunity to maximise their reproductive success, and females may choose mates from only a select group of males, which can vary in heterozygosity across enclosures and across years. We investigated whether female mate choice was for the most heterozygous male per enclosure (each male is compared to the remaining males in their enclosure), or alternatively, if female mate choice had a heterozygosity threshold (regardless of heterozygosity ranking per enclosure, all individuals with a certain level of homozygosity are generally not chosen as mates). To investigate this question, we ran the same model previously described for male reproductive success, however we standardised male internal relatedness within enclosure (referred to herein as “s.IR”). This was calculated as the difference between a males individual IR and the mean IR for all males within the enclosure (i.e. s.IR = IR − IR_enclosure mean_) (following^64^).

### Survival Analysis

A Cox proportional hazard model was used to investigate the effect of inbreeding on survival, with the R package *survival* ^65^. This analysis allows for the inclusion of animals that outlive the study period. Age of death was recorded for all Tasmanian devils that died during the study period. Tasmanian devils were censored if they were still alive at 1 March 2015 (study end date), with their age recorded. The covariates of IR and sex were fitted as predictors of survival. The null expectation of hazard rate (HR) is equal to 1, where the hazard of dying increases with estimates >1, and survival probability increases with estimates <1.

## Results

We found no evidence of null alleles and no significant deviations from Hardy-Weinberg (Table 2). Missing data was low: out of the 200 Tasmanian devils included in this study, ≥95% were genotyped for ≥90% of the 33 markers. Scoring errors based on regenotyping of approximately 16.5% of individuals were minimal (0.2%). The mean number of alleles per locus was 4.00 (range 2–9, n = 33 loci, 200 devils). Mean observed heterozygosity was 0.447 ± (SD) 0.167, and mean expected heterozygosity was 0.459 ± (SD) 0.170 (Table 2).

Internal relatedness (IR) ranged from −0.326 (more heterozygous) to 0.716 (more homozygous) (mean = 0.026 ± 0.188 SE, n = 200). We detected statistically significant evidence of identity disequilibrium in our molecular dataset using both the *g*_2_ analysis (*g*_2_ = 0.011, SD = 0.006, p-value = 0.002) and HHC (r = 0.212, 95% CI = 0.047–0.372). Together these statistics indicate that the genotyped markers are informative of inter-individual variation in inbreeding levels.

### Pedigree reconstruction

All 118 joeys born during 2011–2014 were successfully assigned parentage using joint LOD scores for all candidate parents. Cohabitation was found to be true of the most likely parent-pair combination for 100% of the joeys examined. Incorporating full pedigree information from the insurance population, we observed that 98.6% of individuals in our study population had a pedigree derived inbreeding coefficient of 0.00, with only three greater than 0 (0.03, 0.11 and 0.25) (mean = 0.002 ± SD 0.020, n = 200). As variance is required in the degree of inbreeding to investigate associations among pedigree-based inbreeding and fitness, the pedigree data was uninformative and not analysed further^20^.

### Heterozygosity-fitness correlations

After model averaging, internal relatedness (IR) appeared in the top model or a model within 2 AIC_C_ of the top model for testes volume and male reproductive success (Table 3). Using standardised IR for males gave broadly similar results to absolute IR (Table 3). Standard errors were large for IR in models where it was included (confidence intervals encompassed zero) and relative importance values of IR were low (0.27–0.71; Table 3). For the remaining fitness components (weight at weaning, ulna length, asymmetry and female reproductive success) IR was not included in the top model, nor a model within 2 AIC_C_ of the top model. Of the 97 individuals with weaning weight data only 79 had IR values for joey IR, maternal IR and paternal IR, the remaining were excluded from the model. Missing data comprised of two joey IR values, nine maternal IR values and seven paternal IR values. As missing data was found across all three IR categories, we proceeded to keep our data restricted to joeys that had IR values across all three categories.

**Table 3:** Summary of standardised predictors and their relative importance after averaging of top models (all models within 2AIC_C_) (see methods for details on predictors in each global model).

Response variable	Predictor variables*	Coefficient	SE^†^	CI 95% L	CI 95% U	RI^‡^
Ulna	Intercept	12.45	0.05			
	**Age**	**0**.**45**	**0**.**10**	**0**.**25**	**0**.**65**	**1**.**00**
	**Sex**	**1**.**00**	**0**.**10**	**0**.**80**	**1**.**20**	**1**.**00**
Asymmetry	Intercept	0.14	0.03			
Testes volume	Intercept	11.19	0.81			
	Internal relatedness	0.87	0.48	−0.07	1.81	0.71
	Ulna	0.57	0.60	−0.61	1.75	0.26
	**Body weight**	**3**.**65**	**0**.**56**	**2**.**55**	**4**.**75**	**1**.**00**
Weight at weaning	Intercept	4.54	0.23			
	**Age at weighing**	**0**.**81**	**0**.**17**	**0**.**65**	**0**.**98**	**1**.**00**
	Pen total	−0.34	0.26	−0.60	0.08	0.28
	**Sex**	**0**.**40**	**0**.**14**	**0**.**26**	**0**.**54**	**1**.**00**
Female reproductive success	Intercept	−0.67	0.19			
	**Age**	−**1**.**56**	**0**.**54**	−**2**.**62**	−**0**.**50**	**1**.**00**
Male reproductive success	Intercept	−1.68	0.43			
	Internal relatedness	−0.24	0.54	−1.30	0.82	0.29
	Age	−0.15	0.45	−1.03	0.73	0.23
	**Pen total**	−**1**.**81**	**0**.**43**	−**2**.**65**	−**0**.**97**	**1**.**00**
Standardised male reproductive success	Intercept	−1.68	0.43			
	Internal relatedness	−0.20	0.51	−1.20	0.80	0.27
	Age	−0.16	0.45	−1.04	0.72	0.24
	**Pen total**	−**1**.**79**	**0**.**79**	−**3**.**34**	−**0**.**24**	**1**.**00**

*Standardised predictors to a mean of 0 and a standard deviation of 0.5; all bold predictor variables have confidence intervals that do not include zero.

^†^SE; standard error.

^‡^RI; relative importance.

In our final models, fitness traits were largely influenced by the sex and age of individuals (these parameters had a relative importance value of 1 for several responses) (Table 3). We observed effects of sex on ulna length (males were larger, while controlling for age) and weight at weaning (males were larger, while controlling for age) (Table 3). Unsurprisingly, older individuals showed longer ulna measurements (Table 3). Female reproductive fitness declined with age, but male reproductive fitness did not (Table 3).

The survival hazard rate analysis indicated that there is an imprecise increased risk of the event “death” occurring, with increasing IR. This result was not statistically significant (the confidence interval for this analysis includes the null hypothesis, 1 Table 4).

**Table 4:** Cox proportional hazard model results on the effect of internal relatedness (IR) and survival in the Tasmanian devil.

Predictor	Effect (HR*)	SE	95% CL
Internal Relatedness	1.437	0.695	0.425–4.852
Sex	1.679	0.595	0.989–2.849

*HR; Hazard rate (see methods for details).

## Discussion

We successfully assigned all offspring (using molecular data) to their respective parents and reconstructed a pedigree for a component of the Tasmanian devil insurance population. We investigated the effect of inbreeding on six fitness measures in this population and surprisingly, found no evidence for inbreeding depression. While the pedigree based values showed negligible evidence of inbreeding (98.6% of inbreeding coefficients = 0.00, with only three individuals having an F > 0.00), our HHC and *g*_*2*_ analyses based on microsatellite genotyping revealed statistically significant variation in inbreeding in the Tasmanian devil insurance population. Inbreeding coefficients derived from our molecular data were consistent with trapping proximity (see map^3^) which supports suggestions by Hogg *et al*.^3^ that some original founders in the insurance population may have been closely related individuals. Understanding these potential relationships is beyond the scope of this study but is being addressed by others within our research group. In comparison to a simulation-based study^66^ that evaluated the effectiveness of *g*_*2*_ for estimating the variation in inbreeding, the significant *g*_*2*_ value we report here fell within the range indicated as sufficient to detect correlations between survival and inbreeding^66^. Our results highlight the importance of using both pedigree and molecular tools for population management. A molecular-based approach can provide valuable information in regards to inbreeding that may not necessarily be reflected by a pedigree-based approach (e.g. ref. 67) or when a pedigree is unavailable. Molecular tools can also provide valuable information about individual relationships, such as parentage, which can be used to strengthen pedigree based management and breeding strategies going forward (e.g. ref. 68).

We used a range of life history and morphological traits in an attempt to maximise the possibility of detecting inbreeding depression in the Tasmanian devil, if it is occurring. Indirect fitness measures (both morphometric and physiological) generally experience stabilizing selection pressure toward an optimum, which can create a non-linear relationship between the fitness measurement and heterozygosity^19, 21^. In contrast, direct fitness measures are generally under directional selection and involve a greater number of loci than morphometric and physiological measures^21, 29^. These two contrasting forms of selection can influence the detectability and strength of HFCs, where there is a greater likelihood of detecting HFCs in direct fitness measurements.

None of the direct or indirect fitness measures we recorded showed a strong association with IR in captive Tasmanian devils. There are several explanations for this pattern: (1) this population of Tasmanian devils exhibits little variation in inbreeding (e.g. all individuals have high, or low, levels of inbreeding with little-to-no variance), (2) variation in inbreeding is high, but is not adequately captured by the markers used in this study, or (3) inbreeding has only a weak effect on the fitness traits we measured, in this population. We have shown evidence that there is variance in inbreeding, as our *g*_*2*_ and HHC results were both significant, indicating our genetic dataset does reflect inter-individual variation in inbreeding and thus has the potential to detect inbreeding depression if it occurs (negating arguments 1 and 2, above). Our results may reflect the potential for environmental effects in captivity to lessen inbreeding depression, i.e. reduce the variation in fitness between more/less inbred individuals, and our dataset may have insufficient power to detect such a weak effect.

Given that our molecular dataset has the capacity to detect inbreeding depression if it occurs, we feel that environmental effects may be a plausible explanation for our results in the Tasmanian devil. The effects of inbreeding depression can be decreased in benign environments relative to stressful environments^69, 70^. For example, environmental variation, which may act as a stressor, can differ between a species’ wild environment (generally more variable/stressful) and its captive environment (generally more consistent/benign). Armbruster & Reed (2005) conducted a meta-analysis of 34 studies investigating inbreeding-environment interactions and found an average fitness reduction of 69% in populations exposed to stressful environments relative to benign environments. Thus, inbreeding depression may be weaker in captive conditions, such as the population included in the current study, and may explain the minimal effect we observed between internal relatedness and trait variation^71^. Management practices for the institution in this study involves quarterly health checks (dental examination, wound treatment and parasitic treatment), a feeding regime that is directly proportional to the number of individuals housed in each enclosure, and an assortment of differing enclosure sizes with varying ratios of males and females (breeding facility manager, *pers*. *comm*.). Longevity, resource competition and reproductive competition can all differ in captivity relative to the wild.

A particular environmental mechanism that may impact our results is resource excess, which may mask any signs of inbreeding depression that would be detected under stressful conditions^72^. In general, parental investment can be influenced by resource availability, indirectly impacting reproductive success (e.g. refs 37 and 73). Tasmanian devils give birth to highly altricial young that undergo an intense period of growth in the mother’s pouch^74^. From February to September (breeding season and pouch young), Tasmanian devils at the breeding facility in this study are fed excess (25% increase) resources to accommodate for pouch young investment (breeding facility manager, *pers*. *comm*.). Minimising resource competition among females in this captive population could mask the effects of competitive resource investments, explaining our observation that other measures, such as age, are better predictors of reproductive success than IR. This suggests that there is room for between-individual variation when the effect is large enough (the effect of biological aging) as opposed to small effect predictors (such as internal relatedness). Contrasting to females, resource investment plays a lesser role in male reproductive success, as male marsupials have negligible direct input to their offspring after fertilisation^74^. Tasmanian devil courtship behaviour is not well documented, but females do display both aggressive and submissive behaviours towards potential mates^75^. Forced copulation due to the larger size of male Tasmanian devils (average weight 8.46 kg [current study]) relative to female Tasmanian devils (average weight 7.27 kg [current study]) may result in males acquiring reproductive partners for reasons other than their own IR (e.g. body weight). Furthermore, the majority of Tasmanian devils in the current study were of optimal weight due to resource excess. As such, both low IR males and high IR males have equal opportunity for obtaining recourses, resulting in equal opportunity of acquiring a reproductive partner. For the Tasmanian devil, the full impact of inbreeding depression may only be fully realised under wild conditions.

### Future Directions and Management Implications

We reconstructed a pedigree and found minimal variation in inbreeding coefficients, whereas our molecular data indicated significant variation in underlying identity by descent (inbreeding). Our results suggest that some of the founders of the insurance population may have been closely related, as pairings informed by the shallow pedigree have apparently resulted in some mating among relatives. Our molecular observations of inbreeding, in the absence of pedigree inbreeding, highlight the need for future conservation programs to ensure that the relationships among founding individuals from small, fragmented populations are well understood prior to the commencement of a large-scale insurance population. Incorporating such data will ensure that any potential inbreeding is minimised and accounted for in future breeding management decisions. These considerations are of particular consequence to insurance populations that engage in re-wilding and genetic rescue programs early after establishment, such as the Tasmanian devil insurance population.

Until recently, obtaining an accurate genome-wide estimate of identity-by-descent (IBD) with molecular technology was difficult. However up-to-date research reports that molecular measures of inbreeding can be as accurate, and in some cases, more accurate than pedigree estimates^22, 76^. Between-sibling variation in IBD is not reflected in pedigree-derived inbreeding values (which represent only ‘expected’ values), but can be detected using a sufficiently large panel of molecular markers. The integration of pedigree and molecular assessments will benefit captive population management (with respect to revealing founder relationships), and can be further applied to wild populations in the absence of any pedigree data.

Despite significant variation in inbreeding, we found no evidence for inbreeding depression in the Tasmanian devil; we propose that the benign environment in captivity may attenuate inbreeding depression. Tasmanian devils from the insurance population are already being released to both wild and semi-wild areas (peninsula and island sites)^2^ and we plan to measure their fitness following release. Future work will enable us to test the hypothesis that highly inbred Tasmanian devils experience a fitness disadvantage when released into the wild.

## Electronic supplementary material

Supplementary data
