# Supplementary material for: No evidence of inbreeding depression in a Tasmanian devil insurance population despite significant variation in inbreeding
Source: Sci Rep. 2017 May 12;7:1830. doi: 10.1038/s41598-017-02000-y (PMC5431960; doi:10.1038/s41598-017-02000-y)
Supplement: Supplementary file 1 — Supplementary data [file 41598_2017_2000_MOESM1_ESM.pdf]

No evidence of inbreeding depression in a Tasmanian devil insurance population despite significant variation within inbreeding

Gooley, Rebecca[1]; Hogg, Carolyn J.[1,2]; Belov, Katherine[1]*; Grueber, Catherine[1,3]*
* contributed equally

[1] School of Life and Environmental Sciences, University of Sydney, NSW, 2006, Australia
[2] Zoo and Aquarium Association Australasia, Mosman, NSW, 2088, Australia
[3] San Diego Zoo Global, PO Box 120551, San Diego, CA, 92112, USA

Supplementary Methods

Specific measurement methods are as follows:

1. Ulna length: measured on both mature and immature Tasmanian devils, to the nearest 0.1mm using metal calipers. Both right and left ulna lengths were measured, however for this trait we focused only on the left ulna measurement. Measurements were made in duplicate and averaged for analysis. Measured in December 2014, pre- breeding season.
2. Asymmetry: difference, to the nearest 0.1mm, between the left and right ulna measurements. Measured on both mature and immature Tasmanian devils using metal calipers. Left and right ulnas were measured in duplicate; asymmetry was calculated between the averaged values as left minus right measurements, so that directionality was maintained. Measured in December 2014, pre- breeding season.
3. Testes volume: measured on both mature and immature male Tasmanian devils, to the nearest 0.1mm using metal calipers. Three measurements were taken in duplicate: length and height of the left testis and combined-width of both testes. Volume was calculated using the Lambert equation ($V = L \times W \times H \times 0.71$)[40]. Measured in December 2014, pre- breeding season.
4. Weight at weaning: weaning for the Tasmanian devil is 1 year of age. Weight measurements (to the nearest 0.1kg) for all 118 joeys born from 2011 to 2014 were available at this time point ± 2 months. 41 individuals had two weight entries recorded during the 4-month weighing period and were therefore included twice (see below). Joeys that were hand-raised by keepers were excluded (*n* = 10).
5. Reproductive Success: Reproductive success was measured on an annual basis (2011, 2012, 2013, 2014), as opposed to total reproductive output for an individual. This is to account for the variable number of breeding opportunities an individual experienced, as provided by the species management team at ZAA.
6. Survival: all naturally occurring deaths that were recorded during the period of 2011 to 2014. Survival was recorded to the nearest month.

Supplementary Table S1: Characteristics of the 15 microsatellites found to be monomorphic after screening across 12 Tasmanian devils

Locus (Chromosome)	Primer sequences (5'-3')	Repeat motif	Repeat Length
**Sha002** (Chr 2)	F-GGCTGCCAAGTCACTTAACC<br>R-GAAGTAGTCCTTGAAAGGAG	$(GA)_{12}AA(GA)_{12}$	143
**Sha004** (Chr 1)	F-AGGACACAAAGGAAATCTC<br>R-GGGATGTCATAATGTGAGTG	$(TC)_{31}$	142
**Sha005** (Chr 2)	F-CAGCAGAACATCAGGGAGGA<br>R-AGTTGGGAGCAGAATTAAAG	$(AC)_{20}$	324
**Sha007** (Chr 2)	F-TGTCTAGTTGCCAGGGTCCT<br>R-CTGCCACTTCAAAGTAACAT	$(TG)_{11}AG(TG)_5$	265
**Sha016** (Chr 3)	F-ATAACAACTTCCTCATAGAG<br>R-GGTCATGAAGAATTGGACAC	$(GT)_{21}AT(GT)_4$	158
**Sha017** (Chr 3)	F-TAAGTGTGAGCCTGTTTGAG<br>R-GGCCAATCATTTAGCTTCTC	$(TC)_{15}$	293
**Sha018** (Chr 3)	F-ATGCTATCATATCCTTTCTC<br>R-TAAGGAATATGAGATTACAG	$(AC)_{13}$	251
**Sha019** (Chr 3)	F-TTTGAATTGGGTGATAACAG<br>R-ACAACTCTTTAACTCTTACC	$(AT)_{19}$	194
**Sha021** (Chr 3)	F-ATTAATAGATAGGTTATAGG<br>R-GGAAACTTATTACAATTCAC	$(AT)_2AC(AT)_{18}$	295
**Sha022** (Chr 3)	F-AAAGACAGAGTATAGTAAGA<br>R-ATATATGAATGTGTGAATGT	$(TA)_{22}$	220
**Sha027** (Chr 5)	F-TGCAGTTTACATACTTAGAG<br>R-TTTGTATTCATTGTAATGTC	$(TG)_{18}$	350
**Sha029** (Chr 5)	F-CAGCCCTAGATGTTTATCAG<br>R-CCTAGTAGTGGTATTGACAG	$(GT)_{15}$	334
**Sha031** (Chr 6)	F-AATACCAAACTCCATGAAGG<br>R-TTTGCTGGTTCCACTACTCC	$(GT)_{13}$	353
**Sha035** (Chr 6)	F-CTGACAGAAATCAAAGTCTC<br>R-GGTTTCCTTCAAGTATTAGC	$(AC)_{16}$	344
**Sha038** (Chr4)	F-CACCTTGCATCTAGTTTACA<br>R-CAGAGGGATACATAAGAGTG	$(AC)_{15}$	141

Supplementary Table S2: Summary of global modals, and all predictors that were included in each global model

Response	Internal Relatedness	Ulna	Sex	Body weight	Pen Total (Breeders)[1]	Pen ID	Age[2]
Ulna Length	✓	NA	✓	NA	NA	✓	✓
Asymmetry	✓	✓	✓	NA	✓	✓	NA
Testes Volume	✓	✓	NA	✓	NA	✓	NA
Weight at Weaning	✓	NA	✓	NA	✓	✓	NA
Female Reproductive Success (#/4)	✓	NA	NA	NA	✓	✓	✓
Male Reproductive Success (#/joey pen total)	✓	NA	NA	NA	✓	✓	✓
Standardised Male Reproductive Success (s.IR)	✓	NA	NA	NA	✓	✓	✓
	Age of weighing	Maternal IR[7]	Paternal IR[7]	Individual ID[3]	Year[5]	Litter ID[6]	Litter Size
Ulna Length	NA	NA	NA	NA	NA	NA	NA
Asymmetry	NA	NA	NA	NA	✓	NA	NA
Testes Volume	NA	NA	NA	NA	NA	NA	NA
Weight at Weaning	✓	✓	✓	NA	✓	✓	✓
Female Reproductive Success (#/4)	NA	NA	NA	✓	✓	NA	NA
Male Reproductive Success (#/joey pen total)	NA	NA	NA	✓	✓	NA	NA
Standardised Male Reproductive Success (s.IR)	NA	NA	NA	✓	✓	NA	NA

Check-mark indicates predictors included in the global model; NA indicates predictors not applicable to a particular response (see additional footnotes below); fixed factors are shaded pink; random factors are shaded blue, s.IR is standardised internal relatedness.

1. Pen total of natal/breeding pen – this predictor was included only when there was sufficient sample information for each model.
2. Age and Ulna were highly correlated; so we included "Ulna" in our morphological trait models (asymmetry and testes volume).
3. Individual ID was only included as a random factor when multiple measurements of a single individual were included in the dataset.
4. These measurements were only taken in one year.
5. Year was included as a random factor when measurements occurred over multiple years, to account for annual variation.
6. Litter ID and Litter size were only available to be included for "weight at weaning".
7. Maternal and paternal IR were only available with sufficient sample sizes for "weight at weaning"

Supplementary Table S3: Top model set (top 2AIC$_c$) of generalised linear mixed models for ulna length. All models were fitted with a random factor "pen ID". The final model is provided in Table 3.

Model Statement	df	AIC$_C$	Δ_i^a	w_i^b
β_0 + sex + age	5	141.52	0.00	0.92

df = degrees of freedom

[a] Change in AIC$_C$ from the best model

[b] Akaike model weight

Supplementary Table S4: Top model set (top 2AIC$_c$) of generalised linear mixed models for asymmetry. All models were fitted with a random factor "pen ID" and "year". The final model is provided in Table 3.

Model Statement	df	AIC$_C$	Δ_i^a	w_i^b
β_0	4	-91.60	0.00	0.76

df = degrees of freedom

[a] Change in AIC$_C$ from the best model

[b] Akaike model weight

Supplementary Table S5: Top model set (top 2AIC$_c$) of generalised linear mixed models for testes volume. All models were fitted with a random factor "pen ID". The final model is provided in Table 3.

Model Statement	df	AIC$_C$	Δ_i^a	w_i^b
β_0 + IR + weight	5	166.96	0.00	0.39
β_0 + weight	4	167.81	0.85	0.25
β_0 + IR + ulna + weight	6	168.01	1.05	0.23

df = degrees of freedom

[a] Change in AIC$_C$ from the best model

[b] Akaike model weight

Supplementary Table S6: Top model set (top 2AIC$_c$) of generalised linear mixed models for weight at weaning. All models were fitted with random factors "pen ID", "litter ID" and "year". The final model is provided in Table 3.

Model Statement	df	AIC$_C$	Δ_i^a	w_i^b
β_0 + sex + AAW	7	191.36	0.00	0.26
β_0 + sex + AAW + pen total	8	193.23	1.87	0.10

df = degrees of freedom

[a] Change in AIC$_C$ from the best model

[b] Akaike model weight

AAW = age at weighing

Supplementary Table S7: Top model set (top 2AIC$_c$) of generalised linear mixed models for female reproductive success. All models were fitted with random factors "pen ID", "Individual ID" and "year". The final model is provided in Table 3.

Model Statement	df	AIC$_C$	Δ_i^a	w_i^b
β_0 + age	5	210.60	0.00	0.55

df = degrees of freedom

[a] Change in AIC$_C$ from the best model

[b] Akaike model weight

Supplementary Table S8: Top model set (top 2AIC$_c$) of generalised linear mixed models for male reproductive success. All models were fitted with random factors "pen ID", "Individual ID" and "year". The final model is provided in Table 3.

Model Statement	df	AIC$_C$	Δ_i^a	w_i^b
β_0 + pen total	5	235.52	0.00	0.35
β_0 + IR +pen total	6	236.49	0.97	0.22
β_0 + age + pen total	6	236.97	1.45	0.17

df = degrees of freedom

[a] Change in AIC$_C$ from the best model

[b] Akaike model weight

Supplementary Table S9: Top model set (top 2AIC$_c$) of generalised linear mixed models for standardised male reproductive success. All models were fitted with random factors "pen ID", Individual ID" and "year". The final model is provided in Table 3.

Model Statement	df	AIC$_C$	Δ_i^a	w_i^b
β_0 + pen total	5	235.52	0.00	0.36
β_0 + pen total + s.IR	6	236.73	1.21	0.20
β_0 + pen total + age	6	236.97	1.45	0.18

df = degrees of freedom

[a] Change in AIC$_C$ from the best model

[b] Akaike model weight
